# Photocatalytic Anaerobic Oxidation of Aromatic Alcohols Coupled With H_2_ Production Over CsPbBr_3_/GO-Pt Catalysts

**DOI:** 10.3389/fchem.2022.833784

**Published:** 2022-03-15

**Authors:** Taoran Chen, Mengqing Li, Lijuan Shen, Maarten B. J. Roeffaers, Bo Weng, Haixia Zhu, Zhihui Chen, Dan Yu, Xiaoyang Pan, Min-Quan Yang, Qingrong Qian

**Affiliations:** ^1^ College of Environmental Science and Engineering, Fujian Key Laboratory of Pollution Control & Resource Reuse, Fujian Normal University, Fuzhou, China; ^2^ CMACS, Department of Microbial and Molecular Systems, Leuven, Belgium; ^3^ Hunan Key Laboratory of Nanophononics and Devices, School of Physics and Electronics, Central South University, Changsha, China; ^4^ State Key Lab of Photocatalysis on Energy and Environment, College of Chemistry, Fuzhou University, Fuzhou, China; ^5^ College of Chemical Engineering and Materials, Quanzhou Normal University, Quanzhou, China

**Keywords:** perovskite, CsPbBr3, graphene oxide, anaerobic oxidation of aromatic alcohols, H2 production, photocatalysis

## Abstract

Metal halide perovskites (MHPs) have been widely investigated for various photocatalytic applications. However, the dual-functional reaction system integrated selective organic oxidation with H_2_ production over MHPs is rarely reported. Here, we demonstrate for the first time the selective oxidation of aromatic alcohols to aldehydes integrated with hydrogen (H_2_) evolution over Pt-decorated CsPbBr_3_. Especially, the functionalization of CsPbBr_3_ with graphene oxide (GO) further improves the photoactivity of the perovskite catalyst. The optimal amount of CsPbBr_3_/GO-Pt exhibits an H_2_ evolution rate of 1,060 μmol g^−1^ h^−1^ along with high selectivity (>99%) for benzyl aldehyde generation (1,050 μmol g^−1^ h^−1^) under visible light (*λ* > 400 nm), which is about five times higher than the CsPbBr_3_-Pt sample. The enhanced activity has been ascribed to two effects induced by the introduction of GO: 1) GO displays a structure-directing role, decreasing the particle size of CsPbBr_3_ and 2) GO and Pt act as electron reservoirs, extracting the photogenerated electrons and prohibiting the recombination of the electron–hole pairs. This study opens new avenues to utilize metal halide perovskites as dual-functional photocatalysts to perform selective organic transformations and solar fuel production.

## Introduction

The selective oxidation of alcohols to carbonyls represents one of the most important reactions in both the fine chemical industry and laboratory research (Shibuya et al., 2011; Yang and Xu, 2013; Sharma et al., 2016; Xue Yang et al., 2017; Liu et al., 2018a; Huang et al., 2018; Li et al., 2020; Shang et al., 2021); the carbonyl products are widely used intermediates and precursors for the manufacture of perfumes, pharmaceuticals, and dyes (Liu et al., 2015; Agosti et al., 2020; Xia et al., 2020; Shang et al., 2021). Generally, the oxidative dehydrogenation of alcohols is carried out in the presence of chemical oxidants such as iodine, manganese, chromium oxide, or molecular oxygen. The utilization of costly and toxic chemical agents not only results in the production of stoichiometric amounts of waste but also often generates overoxidized products (Mallat and Baiker, 2004; Lang et al., 2014; Meng et al., 2018a; Meng et al., 2018b; Kampouri and Stylianou, 2019; Crombie et al., 2021; Shang et al., 2021). Particularly, the removed protons are consumed by the oxidant in these strategies resulting in the loss of a potentially interesting source of hydrogen gas (Han et al., 2020; Wang et al., 2021). In this respect, if the hydrogen atoms released from the alcohols during oxidation can be converted into H_2_, that is, combining the dehydrogenation reaction with H_2_ evolution, it would not only improve the atom economy of the reaction and the added value of the products but also provide a revolutionary technology for H_2_ production. However, coupling the oxidative dehydrogenation of alcohols with reductive hydrogen production is challenging.

Within this context, the advancement of photocatalytic anaerobic oxidation technology in recent years provides a promising strategy. This approach utilizes photogenerated holes to oxidize organics while employing photoelectrons to reduce the removed protons to produce H_2_, thus completing the oxidative–reductive coupled reaction (Weng et al., 2016; Han et al., 2017; Zhou et al., 2020; Peixian Li et al., 2021). Different from traditional photocatalytic aerobic oxidation, the oxygen-free condition effectively inhibits the consumption of the removed protons to produce water and avoids the formation of strong oxidation radicals (like superoxide radicals), which is favorable for improving the product selectivity. Theoretically, the anaerobic dehydrogenation coupled to H_2_ evolution is initiated by the oxidation half-reaction to remove protons, which is considered to be a rate-limiting step (Liu et al., 2018b; Wang et al., 2021). As such, to obtain high catalytic efficiency, the efficient separation and migration of holes, that is, the exploration of advanced photocatalytic materials with high hole mobility and long carrier lifetime, to oxidize the organic substrates, is essential.

In recent years, the halide perovskite (ABX_3_) material has been deemed as a promising new-generation photocatalyst alternative due to its remarkable optoelectronic properties such as a large extinction coefficient and an excellent visible light-harvesting ability (Zhao and Zhu, 2016; Xu et al., 2017; Akkerman et al., 2018; Huang et al., 2019; Huang et al., 2020; Wang et al., 2022). Importantly, the halide perovskite with a delocalized energy level exhibits a small hole effective mass (Yuan et al., 2015) and high hole mobility (100 cm^2^ V^−1^ s^−1^), which is hundreds of times higher than traditional semiconductor materials such as TiO_2_ (Wehrenfennig et al., 2014; Bin Yang et al., 2017). Moreover, the perovskite also shows a long carrier lifetime of tens to hundreds of µs and diffusion length of μm levels, providing more opportunities for the diffusion and utilization of photoinduced holes and electrons (Dong et al., 2015; Bi et al., 2016). In this context, these unique features enable the metal halogen perovskite to be an appealing candidate for the organic conversion-coupled hydrogen production reaction, but the research is still rarely reported so far.

Inspired by the foregoing considerations, we herein fabricate CsPbBr_3_/GO-Pt composites for photocatalytic coupling redox reaction. In the composite, the CsPbBr_3_ acts as a photoactive component, while the GO plays an important role in decreasing the particle size of CsPbBr_3_, together with Pt as electron reservoirs to extract photogenerated electrons and prohibit the recombination of electron–hole pairs. By taking selective anaerobic oxidation of aromatic alcohols as model reactions, the as-prepared CsPbBr_3_/GO-Pt shows obvious photoactivity for the simultaneous production of aromatic aldehydes and H_2_. An optimal H_2_ evolution rate of 1,060 μmol g^−1^ h^−1^ along with a benzyl aldehyde production rate of 1,050 μmol g^−1^ h^−1^ is realized over the CsPbBr_3_/1.0% GO-1%Pt composite under visible light irradiation (*λ* > 400 nm). Mechanism study reveals that the carbon-centered radical serves as a pivotal radical intermediate during the photoredox process.

## Experimental Section

### Materials

Cesium bromide (CsBr, 99.999%) and lead bromide (PbBr_2_, 99.0%) were purchased from Macklin. N, N-dimethylformamide (DMF), toluene, graphite powder, potassium persulfate (K_2_S_2_O_8_), phosphorus pentoxide (P_2_O_5_), concentrated sulfuric acid (H_2_SO_4_, 98%), concentrated nitric acid (HNO_3_, 65%), hydrogen peroxide solution (H_2_O_2_, 30%), potassium permanganate (KMnO_4_, 99.5%), tetrabutylammonium hexafluorophosphate (TBAPF_6_, 98%), hydrochloric acid (HCl, 36%), ethanol, acetonitrile, ethyl acetate, acetone, isopropanol, and benzyl alcohol all were obtained from Sinopharm Chemical Reagent Co., Ltd. (Shanghai, China). All the chemicals were used as received without further purification.

### Catalyst Preparation

#### Preparation of Graphene Oxide

GO was synthesized from natural graphite powder using a modified Hummers’ method (Hummers and Offeman, 1958; Yang and Xu, 2013). The details are described in the supporting information.

#### Synthesis of CsPbBr_3_/Graphene Oxide and CsPbBr_3_

CsPbBr_3_/GO was synthesized *via* a well-established anti-solvent precipitation method at room temperature (Huang et al., 2018). In brief, a certain amount of GO (2.5, 5, 7.5, 10 mg) was first dispersed in 10 ml of N, N-dimethylformamide (DMF) by ultrasonication. Then, 1 mmol CsBr and 1 mmol PbBr_2_ were added to the solution. After completely dissolving CsBr and PbBr_2_, the mixture was added dropwise into 80 ml toluene under vigorous stirring, which generated orange precipitation immediately. After that, the precipitation was centrifuged, washed with toluene three times, and then dried in a vacuum oven at 60 °C for 12 h. The blank CsPbBr_3_ was prepared by following the same procedure without the addition of GO.

### Characterizations

Scanning electron microscopy (SEM) images of the samples were characterized by using Hitachi 8100. Transmission electron microscopy (TEM) images were recorded using a 200 kV JEOL-2100f transmission electron microscope. The X-ray diffraction (XRD) patterns of the catalysts were characterized on a Bruker D8 advance X-ray diffractometer operated at 40 kV and 40 mA with Cu Kα radiation in the 2*θ* ranging from 10° to 80°. UV–vis diffuse reflectance spectra (DRS) were obtained on an Agilent CARY-100 spectrophotometer using 100% BaSO_4_ as an internal standard. X-ray photoelectron spectroscopy (XPS) was recorded on Thermo Fisher (Thermo Scientific K-Alpha+) equipped with a monochromatic Al Kα as the X-ray source. All binding energies were referenced to the C 1s peak at 284.8 eV of surface adventitious carbon. Raman spectra were recorded by using a Thermo Fisher-DXR 2xi with a laser at a wavelength of 532 nm. Photoluminescence (PL) measurements were performed on a spectrophotometer (MS3504i) with an excitation wavelength of 405 nm, and time-resolved PL (TRPL) was recorded by using a photon-counting photomultiplier (PMT) (Pico Quant, PMC-100–1).

Electron paramagnetic resonance (EPR) measurements were performed at room temperature using a Magnettech ESR5000 spectrometer. For EPR measurements, 10 mg sample powders were dispersed in a mixed solution of 0.5 ml CH_3_CN containing 10 μL benzyl alcohol (BA) and 2 μL 5,5-dimethyl-1-pyrroline-N-oxide (DMPO). Then, the suspension was injected into a glass capillary, which was further placed in a sealed glass tube under argon (Ar) atmosphere. The sealed glass tube was placed in the microwave cavity of the EPR spectrometer and was irradiated with a 300-W Xe lamp (PLS-SXE 300D, Beijing Perfectlight Technology Co., Ltd.) equipped with a 400-nm cutoff filter during the EPR measurement at room temperature.

### Electrochemical Measurements

All the electrochemical measurements were recorded in a conventional three electrodes cell using a CHI 760E instrument. A platinum wire was used as the counter electrode (CE), and an Ag/AgCl electrode was used as the reference electrode (RE). The electrolyte was ethyl acetate solution containing 0.1 M tetrabutylammonium hexafluorophosphate (TBAPF_6_). The working electrodes were prepared using CsPbBr_3_ and CPB/1.0% GO samples. Typically, the fluorine-doped tin oxide (FTO) substrate was first cleaned by ultrasonication in ethanol and then rinsed with deionized water and acetone for half an hour. Then, 10 mg of the catalyst was dispersed in 1 ml of isopropanol to get slurry. After that, 50 µL of the slurry was spread on the conductive surface of the FTO glass and then dried at 60°C for 2 h to improve adhesion. The exposed area of the working electrode was 1 cm^2^. A 300-W Xe lamp system (PLS-SXE 300D, Beijing Perfectlight Technology Co., Ltd.) equipped with a 400-nm cutoff filter was used as the irradiation source. The electrochemical impedance spectroscopy (EIS) measurements were carried out in a frequency range from 1 Hz to 1 MHz. The photocurrent measurement was performed under visible light irradiation (*λ* > 400 nm) using a 300-W Xenon lamp source (PLS-SXE 300D, Beijing Perfectlight Technology Co., Ltd.).

### Photocatalytic Activity

The photocatalytic H_2_ evolution integrated with aromatic alcohol oxidation was tested in a quartz reactor. Typically, 10 mg of photocatalyst, 0.2 mmol aromatic alcohol, and 1.0% Pt (H_2_PtCl_6_ as a precursor) were added into a quartz reactor containing 3 ml CH_3_CN (purge with Ar gas for 15 min). Then, the reactor was irradiated by visible light (*λ* > 400 nm) using a 300 W Xe lamp (PLS-SXE 300D, Beijing Perfect light Technology Co., Ltd.) under continuous stirring. After the reaction, the gas product was analyzed by a gas chromatograph (GC 9790pLus, Fu Li, China, TCD detector, Ar as the carrier gas). Liquid products were analyzed by gas chromatography (Shimadzu GC-2030, FID detector) after centrifuging the suspension at 10,000 rpm to remove the catalyst. The test conditions for the long-time experiment were similar to the aforementioned description, except that the reaction time was extended to 20 h.

The conversion efficiency of aromatic alcohols (A) and selectivity of aldehydes (AD) production were calculated using the following equations:

$$\mathrm{Conversion}\left(\%\right)=100\times \left[\left(C_{0}-C_{A}\right)/C_{0}\right]\;\%,$$

$$\mathrm{Selectivity}\left(\%\right)=100\times \left[C_{AD}/\left(C_{0}-C_{A}\right)\right]\%$$

where C_0_ is the initial concentration of aromatic alcohols, and C_A_ and C_AD_ are the concentrations of aromatic alcohols and aldehydes measured after the photocatalytic reaction for a specific time, respectively.

## Results and Discussion

**SCHEME 1:**
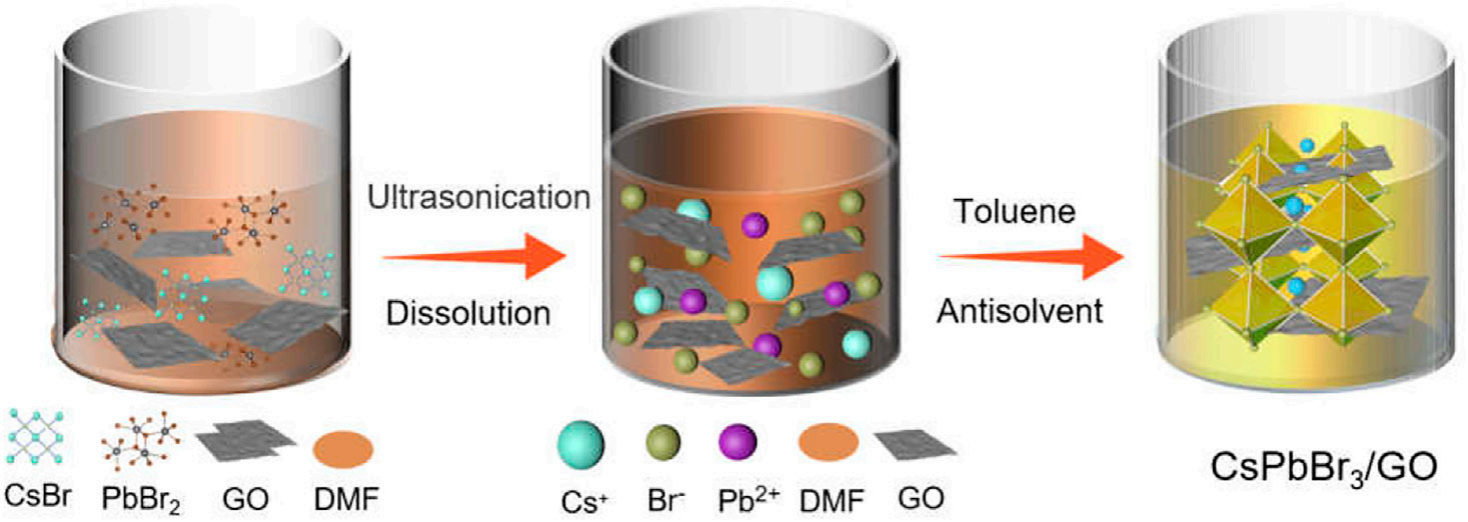
Schematic diagram for the fabrication of CsPbBr_3_/GO composites.

The fabrication of the CsPbBr_3_/GO (denoted as CPB/GO) composite is realized *via* a simple anti-solvent method by adding GO into the precursor solution of CsPbBr_3_ (for more details, please refer to the experimental section), as illustrated in Scheme 1. The crystal structures of the CsPbBr_3_ and CPB/GO composites were analyzed by X-ray diffraction (XRD). As displayed in Supplementary Figure S1A (Supporting Information), for all the as-obtained samples, the main XRD peaks are indexed to the monoclinic CsPbBr_3_ (JCPDS card NO. 00–018–0,364) (Chen et al., 2021). No GO diffraction peaks were observed in the XRD patterns of the CPB/GO samples because of the low weight content (≤1.5%). Raman analysis in Supplementary Figure S1B shows that the as-prepared GO and CPB/GO composite both display two peaks at 1,599 and 1,360 cm^−1^, which belong to the typical D and G bands of GO, respectively (Huang et al., 2021). Moreover, an obvious peak at 308 cm^−1^ assigned to the CsPbBr_3_ was detected in CsPbBr_3_ and CPB/GO (Wang et al., 2020), which verifies the formation of the hybrid composite. Supplementary Figure S1C shows the UV–vis diffuse reflectance spectra (DRS) of blank CsPbBr_3_ and CPB/GO composites. Owing to the addition of GO, the light absorption of CPB/GO composites in the region of visible light (550–800 nm) gradually enhances with the increase in the weight ratios of GO, and the colors of the samples change from yellow to brown (Supplementary Figure S2), which can be attributed to the significant background absorption of GO (Xu et al., 2011). The absorption edges for CsPbBr_3_ and CPB/GO are around 548 nm, which correlates with the intrinsic absorption of the material (Supplementary Figure S3).

**FIGURE 1:**
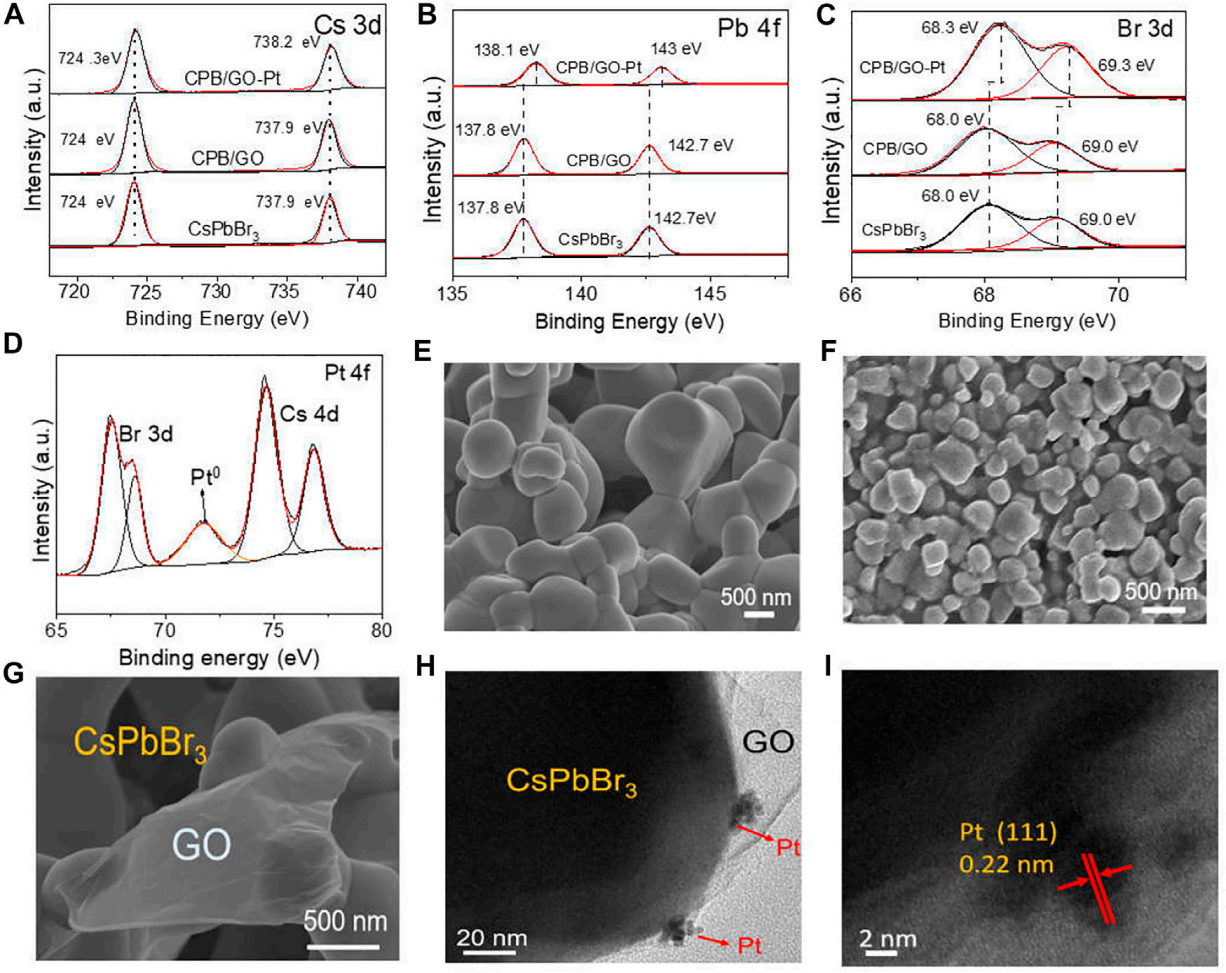
**(A)** Cs 3d, **(B)** Pb 4f, **(C)** Br 3d, and **(D)** Pt 4f XPS spectra of CsPbBr_3_, CPB/GO, and CPB/GO-Pt composites; SEM images of **(E)** blank CsPbBr_3_ and **(F,G)** the CPB/GO composite; **(H)** TEM and **(I)** HRTEM images of the CPB/GO-Pt composite.

Noble metal Pt nanoparticles are further introduced into the CPB/GO composite for enhancing the catalytic performance. The valence states of different elements have been investigated by X-ray photoelectron spectroscopy (XPS). High-resolution C 1s peaks of CPB/GO and CPB/GO-Pt samples in Supplementary Figure S4 show a C–O bond at 286.6 eV and C=O bond at 287.7 eV, which can be ascribed to the introduction of GO. Figure 1A shows the Cs 3d spectra of blank CsPbBr_3_, CPB/GO, and CPB/GO-Pt samples. The double peaks of Cs 3d at 724.1 and 738.1 eV are ascribed to Cs^+^ in CsPbBr_3_ (Jiang et al., 2020; Liang Li et al., 2021), and no obvious change was observed for blank CsPbBr_3_ and CPB/GO, while a positive shift was detected for the CPB/GO-Pt sample. This is attributed to the electron transfer from CsPbBr_3_ to Pt, thus reducing the electron density and altering the coordination environment of Cs. A similar observation can also be made for both the Pb 4f and Br 3d spectra over these samples (Figures 1B, C). The Pt 4f spectrum in Figure 1D exhibited a peak located at 71.6 eV assigned to the Pt^0^ (Qadir et al., 2012), while another peak is the superposition with the Cs 4d peaks, which suggests that Pt is present in metallic state (Wang et al., 2017). The CsPbBr_3_/1.0% GO-1%Pt was characterized by inductively coupled plasma mass spectrometry (ICP-MS), and the detected mass content of Pt is ca. 0.95% (Supplementary Table S1), closely matching the targeted amount (i.e., 1%).

Scanning electron microscopy (SEM) is used to study the morphologic details of blank CsPbBr_3_, CPB/GO, and CPB/GO-Pt samples (Figures 1E–G and Supplementary Figure S5). The average particle size of the CPB/GO composite is much smaller (0.3–0.5 µm) than that of the blank CsPbBr_3_ sample (0.8–1.2 µm) (Supplementary Figure S6A). This can be attributed to the fact that GO with abundant functional groups promotes the nucleation process of CsPbBr_3_, thus producing more seeds and hence leading to the smaller size of final perovskite. The structure-directing role of GO to decrease the size of a semiconductor has been widely reported over graphene-based semiconductor composites (Yang et al., 2014). The enlarged SEM image of the CPB/GO composite in Figure 1G shows an intimate interfacial contact between the GO sheets and the CsPbBr_3_ particles. The CPB/GO-Pt sample features the same morphology as CPB/GO (Supplementary Figure S5), proving the maintenance of the structure during the Pt modification. This has been further verified by TEM analysis. As shown in Supplementary Figures S6B, C, the TEM image of the CPB/GO composite discloses the fact that the CsPbBr_3_ particles have been well linked with or wrapped by GO nanosheets, and Cs, Pb, and Br are homogeneously distributed on the C element (Supplementary Figure S6D). Moreover, Figures 1H, I clearly show that the Pt nanoparticles are loaded onto the surface of CsPbBr_3_ with a lattice fringe of 0.22 nm corresponding to Pt (111), and the size of Pt was calculated to be ca. 3.1 nm (Supplementary Figure S7).

**FIGURE 2:**
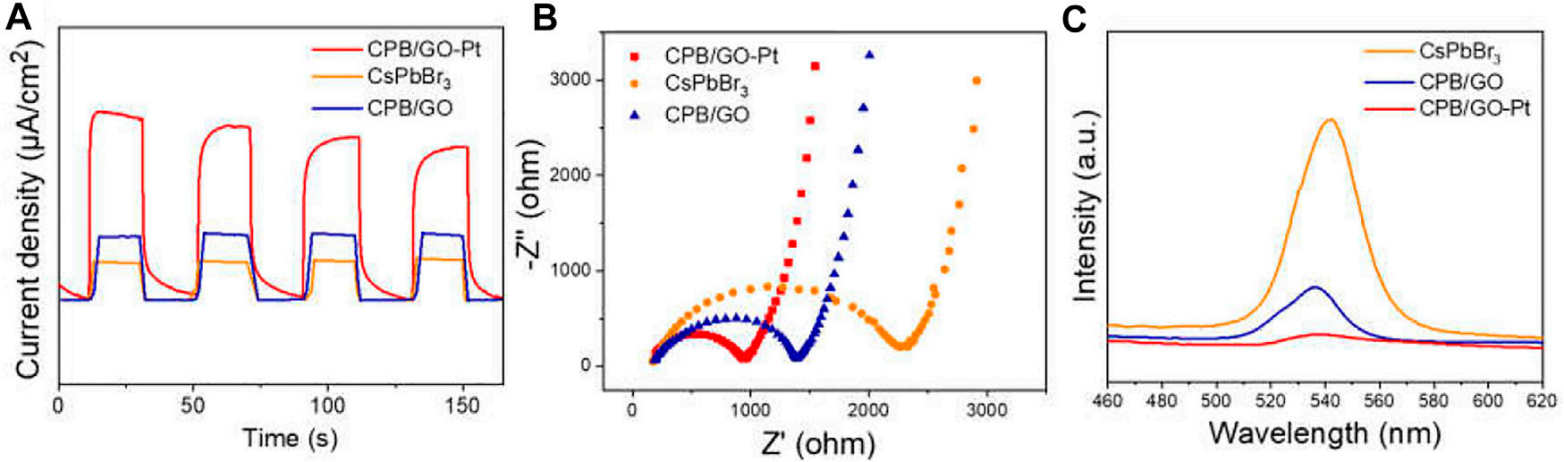
**(A)** Chopped photocurrent responses, **(B)** Nyquist plots, **(C)** PL spectra of CsPbBr_3_, CPB/GO, and CPB/GO-Pt composites.

To further study the influence of the introduction of GO and Pt on charge separation and migration, a series of photoelectrochemical characterizations over blank CsPbBr_3_, CPB/GO, and CPB/GO-Pt composites have been carried out. As shown in Figure 2A, the photocurrent response tests of these samples reveal that the CPB/GO-Pt hybrid composite (taking CPB/1.0% GO-1%Pt with optimal photoactivity as an example) displays higher current density than blank CsPbBr_3_ and CPB/GO samples, indicating a more efficient separation of the photogenerated carrier (Liao et al., 2021). Figure 2B presents the electrochemical impedance spectroscopy (EIS) study of these samples, which is employed to study the charge transfer resistance of the samples. The hybrid CPB/GO-Pt shows the smallest arc diameter among these samples, demonstrating a more efficient charge transfer between the electrode and electrolyte solution over CPB/GO-Pt as compared with CsPbBr_3_ and CPB/GO samples (Lu et al., 2021). This result is consistent with the observation in photocurrent responses tests.

Moreover, photoluminescence (PL) has been performed to investigate electron–hole recombination. As shown in Figure 2C, blank CsPbBr_3_ shows a strong emission peak at 546 nm in the PL spectrum upon excitation with 364-nm electromagnetic waves. For CPB/GO and CPB/GO-Pt composites, the PL intensity is significantly quenched since the radiative recombination of photogenerated electron–hole pairs is diminished due to the electron-accepting nature of GO and Pt (Min-Quan Yang et al., 2017; Chen et al., 2021). This is also supported by the time-resolved photoluminescence (TRPL) decay analysis, as displayed in Supplementary Figure S8 and Supplementary Table S2. The TRPL curve of CPB/GO exhibits a faster decay than that of blank CsPbBr_3_, which can be attributed to the efficient transfer of photogenerated electrons from CsPbBr_3_ to GO sheets at a suitable energy level (Xu et al., 2017; Su et al., 2020). The collective photoelectrochemical analyses consolidate that the integration of GO and Pt with CsPbBr_3_ leads to a more efficient electron–hole separation and rapid charge transfer in the composite, which is critical for boosting the photoactivity (Yang et al., 2019).

Next, the photocatalytic performances of the samples have been evaluated for the anaerobic photocatalytic oxidation of aromatic alcohols coupled with H_2_ production under visible light irradiation (*λ* > 400 nm). Both H_2_ and BAD are not detected in the dark or without the catalyst, indicating that the reaction is driven by a photocatalytic process (Supplementary Figure S9). The samples of GO and GO-Pt mainly serve as cocatalysts since no products are detected during the photocatalytic reaction process. Moreover, the blank CsPbBr_3_ cannot produce any products due to the limited reaction kinetics, while the construction of the CPB/1%GO composite leads to low photoactivity toward H_2_ (90 μmol g^−1^ h^−1^) and BAD (94 μmol g^−1^ h^−1^) generation. The introduction of Pt into CsPbBr_3_ improves the catalytic performance, and the production of BAD and H_2_ are obtained in almost stoichiometric amounts over CsPbBr_3_-1%Pt, indicating a high selectivity (>99%) of the reaction.

**FIGURE 3:**
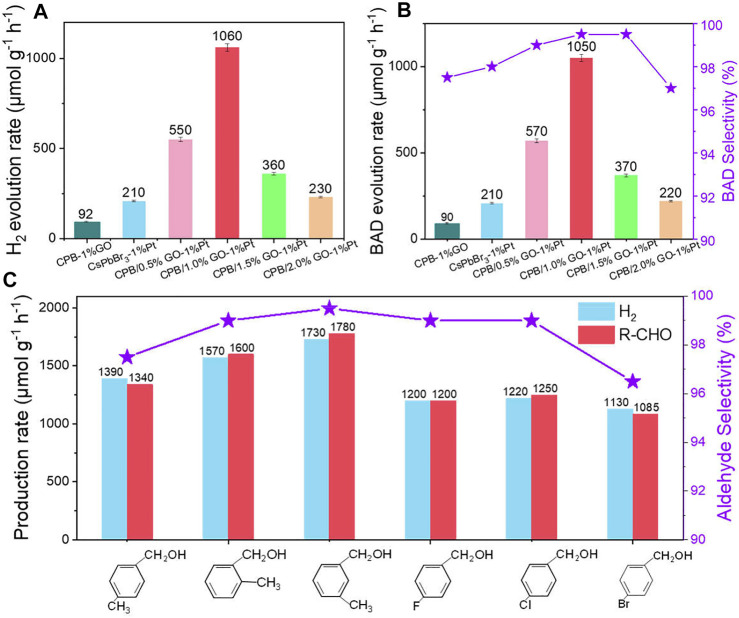
**(A)** Photocatalytic activity of H_2_ evolution, **(B)** BAD evolution and selectivity of CsPbBr_3_-1%Pt and CPB/x% GO-1%Pt with different weight ratios of GO. **(C)** The photocatalytic activities for H_2_ and aldehyde generation of different aromatic alcohols using CPB/1.0% GO-1%Pt as a photocatalyst. Reaction conditions: 10 mg catalyst, 1.0% Pt, 0.2 mmol BA, 3 ml of CH_3_CN, Ar atmosphere, and visible light (λ > 400 nm).

After integration with both GO and Pt, the BAD and H_2_ evolution efficiencies are further enhanced compared with those of CsPbBr_3_-1%Pt and CPB/1%GO. In detail, the optimal photoactivity is obtained on the sample of the CPB/1% GO-1%Pt composite (H_2_ and BAD evolution rates of 1,060 and 1,050 μmol g^−1^ h^−1^_,_ respectively), which is about fivefold as high as that of the CsPbBr_3_-1%Pt sample (Figures 3A,B). This is well in accordance with previous reports stating that loading a suitable amount of GO, which acts as an electron acceptor, with semiconductor photocatalysts can notably improve photoactivity (Chen et al., 2021). Increasing the GO content further to 1.5% resulted in decreased photoactivity. This may be ascribed to the shielding effect of the GO (Yang et al., 2013). On the one hand, the active sites on the surface of CsPbBr_3_ may be blocked due to the addition of high amounts of GO. On the other hand, GO with black color could also absorb the light, which is competed with CsPbBr_3_ and inhibits light passing through the depth of the reaction solution.

Based on the high photocatalytic performance of the CPB/1.0% GO-1%Pt, the photocatalytic anaerobic dehydrogenation of a series of aromatic alcohols with different substituents has been tested. As shown in Figure 3C, moderate H_2_ generation and aldehyde production are obtained for 4-chlorobenzyl alcohol, 4-bromobenzyl alcohol, 4-fluorobenzyl alcohol, 4-methylbenzyl alcohol, 3-methylbenzyl alcohol, and 2-methylbenzyl alcohol. Particularly, for all the substrates bearing electron-donating or electron-withdrawing functional groups, the reactions show high selectivity for aldehyde production (>98%). There is no other byproduct generated during the photocatalytic period, verifying good applicability of the CPB/GO-Pt as a photocatalyst toward solar light–driven integrated organic synthesis and H_2_ evolution.

To assess the stability of the CPB/GO-Pt composite, a long-term photoactivity test has been carried out. As depicted in Supplementary Figure S10, under continuous irradiation for 20 h, the CPB/1.0% GO-1%Pt photocatalyst shows no obvious deactivation with consistent H_2_ and BAD production. The composite material manifests excellent stability of the binary composite. In addition, the morphology and crystal structure of the used CPB/1.0% GO-1% Pt have been investigated by SEM (Supplementary Figures S11 and S12) and XRD (Supplementary Figure S13), and no obvious changes between the used and fresh composites were detected. All these aforementioned results are strong evidence for the good stability of the CPB/GO-Pt composite under the used experimental conditions, which is attributed to the mild polarity of acetonitrile and CsPbBr_3_ substrates that are demonstrated to be stable in this solution (Ou et al., 2018; Zhang et al., 2021).

**FIGURE 4:**
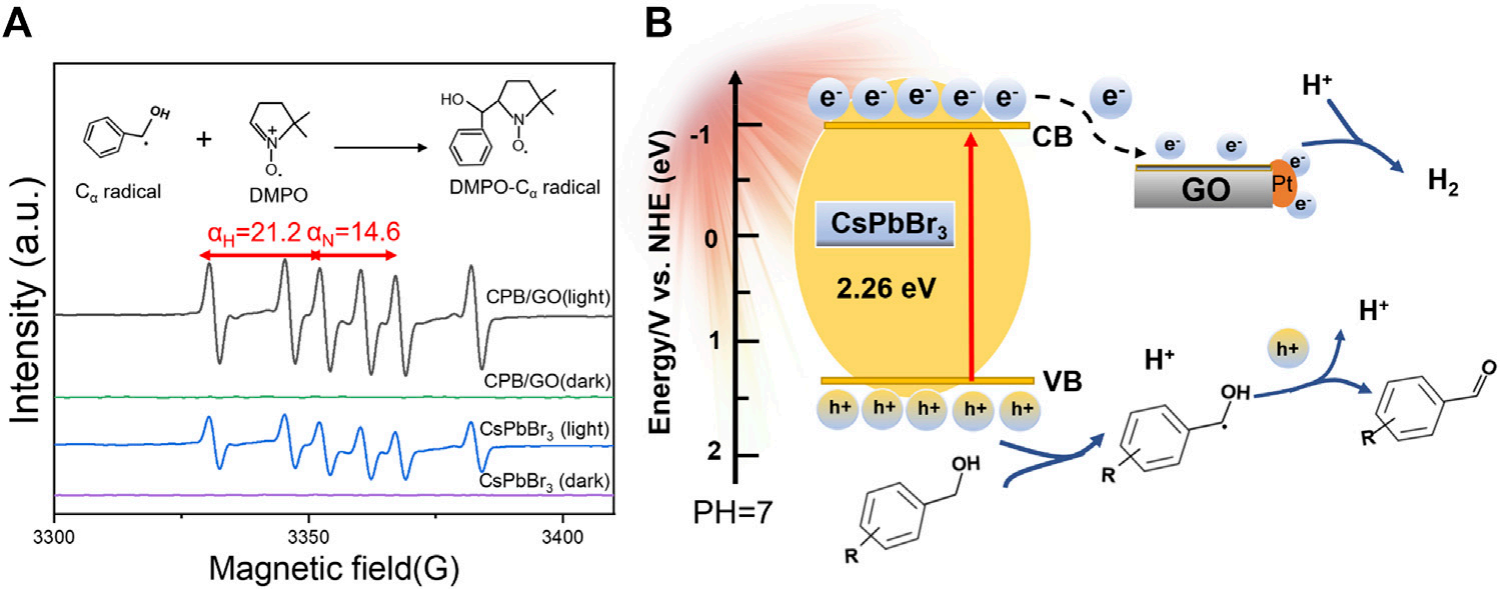
**(A)** *In situ* EPR spectra of CsPbBr_3_ and CPB/GO composites tested in Ar-saturated CH_3_CN solution in the presence of DMPO. **(B)** Schematic illustration of photocatalytic H_2_ production integrated with aromatic aldehyde synthesis.

To further study the radical intermediates involved in the catalytic system, electron paramagnetic resonance (EPR) analysis is performed under visible light using 5,5-dimethyl-1-pyrroline N-oxide (DMPO) as a trapping agent. As presented in Figure 4A, there were no free radical signals in the dark. Under light illumination, six characteristic signal peaks were observed for both CsPbBr_3_ and the CPB/1.0% GO composite, which belongs to the carbon-centered radical adduct (*α*_H_ = 21.2 and *α*_N_ = 14.6, corresponding to the hydrogen and nitrogen hyperfine splitting for the nitroxide nitrogen) (Qi et al., 2020). The signal intensity of DMPO-CH(OH)Ph over the CPB/GO composite is stronger than that of blank CsPbBr_3_, indicating that a larger amount of such carbon-centered radicals was generated in the CPB/GO-catalytic system. This should be ascribed to the enhanced photogenerated charge–transferring ability of CPB/GO in contrast to blank CsPbBr_3_, which increases their likelihood of interaction with the alcohol substrates. The chemical reaction equations for photocatalytic BA oxidation coupled with H_2_ generation over CsPbBr_3_/GO-Pt are presented in Supplementary Figure S14.

On the basis of the aforementioned analyses, a tentative photocatalytic mechanism is proposed for the coupled reaction system toward H_2_ evolution integrated with the conversion of aromatic alcohols to aromatic aldehydes over the CPB/GO-Pt composite. As displayed in Figure 4B, under the illumination of visible light, CsPbBr_3_ in the CPB/GO-Pt composite is excited to generate electrons and holes. Owing to the matched energy level and intimate interfacial contact between CsPbBr_3_ and GO, the electrons tend to migrate from CsPbBr_3_ to GO and Pt, leaving photoinduced holes in the valence band (VB) of CsPbBr_3_. Meanwhile, the holes will attack the C–H bond of absorbed BA to generate CH(OH)Ph radicals and protons (Wu et al., 2018). Then, the CH(OH)Ph radicals can be further oxidized to generate BAD and protons. The abstracted protons from BA are reduced to produce H_2_ by the electrons collected on the surfaces of GO and Pt in the CPB/GO-Pt composite, thus completing the coupled redox reaction.

## Conclusion

In summary, we have realized efficient photocatalytic dehydrogenation of aromatic alcohols for simultaneous aldehyde production and H_2_ evolution over CsPbBr_3_/GO-Pt composite under visible light (*λ* > 400 nm). The results show that optimal amounts of CsPbBr_3_/GO-Pt composite can obtain nearly five times the yield of products (BAD and H_2_) as high as that of CsPbBr_3_-Pt. The enhanced photoactivity of CPB/GO-Pt composite is ascribed to the critical roles of GO in tuning the size of CsPbBr_3_ and together with Pt to extract the photogenerated electrons to boost the migration of photogenerated charge carriers. Furthermore, the carbon-centered radicals have been proven as the pivotal radical intermediate during the photoredox reaction by *in situ* electron paramagnetic resonance (EPR). This work is anticipated to open an avenue for the utilization of halide perovskites as promising candidates in cooperative organic transformation coupling with solar fuel production by the full utilization of photogenerated electrons and holes.

## Data Availability

The raw data supporting the conclusions of this article will be made available by the authors, without undue reservation.
