# Supplementary material for: Photocatalytic Anaerobic Oxidation of Aromatic Alcohols Coupled With H2 Production Over CsPbBr3/GO-Pt Catalysts
Source: Front Chem. 2022 Mar 15;10:833784. doi: 10.3389/fchem.2022.833784 (PMC8965384; doi:10.3389/fchem.2022.833784)
Supplement: Supplementary file 1 [file DataSheet1.docx]

Supporting Information

**Photocatalytic anaerobic oxidation of aromatic alcohols coupled with H_2_ production over CsPbBr_3_/GO-Pt catalysts**

Taoran Chen,^a^ Mengqing Li,^a^ Lijuan Shen,^a^ Maarten B. J. Roeffaers,^b^ Bo Weng,^b,^ * Haixia Zhu,^c^ Zhihui Chen,^c^ Dan Yu,^d^ Xiaoyang Pan,^e^ Min-Quan Yang^a^* and Qingrong Qian^a^

^a^ College of Environmental Science and Engineering, Fujian Key Laboratory of Pollution Control & Resource Reuse, Fujian Normal University, Fuzhou 350007, P. R. China

^b^ cMACS, Department of Microbial and Molecular Systems, KU Leuven, Celestijnenlaan 200F, 3001 Leuven, Belgium

^c^ Hunan Key Laboratory of Nanophononics and Devices, School of Physics and Electronics, Central South University, 932 South Lushan Road, Changsha, Hunan 410083, P. R. China

^d^ State Key Lab of Photocatalysis on Energy and Environment, College of Chemistry, Fuzhou University, Fuzhou, 350116, P. R. China.

^e^ College of Chemical Engineering and Materials, Quanzhou Normal University, Quanzhou 362000, China

*To whom correspondence should be addressed

E-mail: [bo.weng@kuleuven.be](mailto:bo.weng@kuleuven.be); [yangmq@fjnu.edu.cn](mailto:yangmq@fjnu.edu.cn)

**1. Experimental**

Synthesis of graphene oxide (GO): GO was synthesized from natural graphite powder by a modified Hummers’ method. In detail, 2 g of graphite powder (supplied from Sinopharm Chemical Reagent Co., Ltd., China) was put into a mixture of 12 mL of concentrated H_2_SO_4_, 2.5 g of K_2_S_2_O_8_, and 2.5 g of P_2_O_5_. The solution was heated to 80 °C in an oil-bath kept stirring for 24 h. The mixture was then carefully diluted with 500 mL of deionized (DI) water, filtered, and washed until the pH of rinse water became neutral. The product was dried under ambient condition overnight. This pre-oxidized graphite was then subjected to oxidation described as follows. In a typical procedure, pre-oxidized graphite powder was added to a mixture of 120 mL of concentrated H_2_SO_4_ and 30 mL HNO_3_ under vigorous stirring, and the solution was cold to 0 °C. Then, 15 g of KMnO_4_ was added gradually under stirring and the temperature of the mixture was kept to be below 20 °C by cooling. Successively, the mixture was stirred at room temperature for 96 h, and then diluted with 1 L of DI water in an ice bath to keep the temperature below 50 °C for 2 h. Shortly after the further diluted with 1 L of DI water, 20 mL of 30% H_2_O_2_ was then added to the mixture and a brilliant yellow product was formed along with bubbling. The mixture was filtered and washed with 1:10 HCl aqueous solution to remove metal ions followed by DI water to remove the acid. The filter cake was then dispersed in water by a mechanical agitation. Low-speed centrifugation was done at 1000 rpm for 2 min. The supernatant then underwent two more high-speed centrifugation steps at 8000 rpm for 15 min to remove small GO pieces and water-soluble byproduct. The final sediment was redispersed in water with mechanical agitation or mild sonication using a table-top ultrasonic cleaner, giving a solution of exfoliated GO.

**2. Additional data**


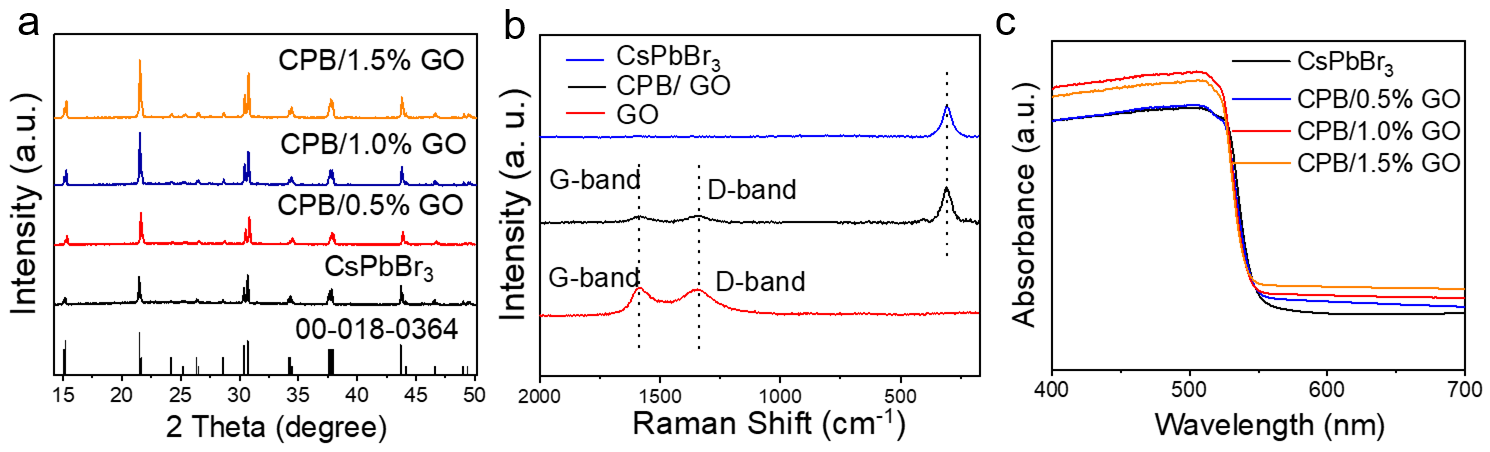


**Figure S1.** a) XRD patterns, b) Raman spectra, and c) DRS spectra of CsPbBr_3_ and CPB/GO composites.


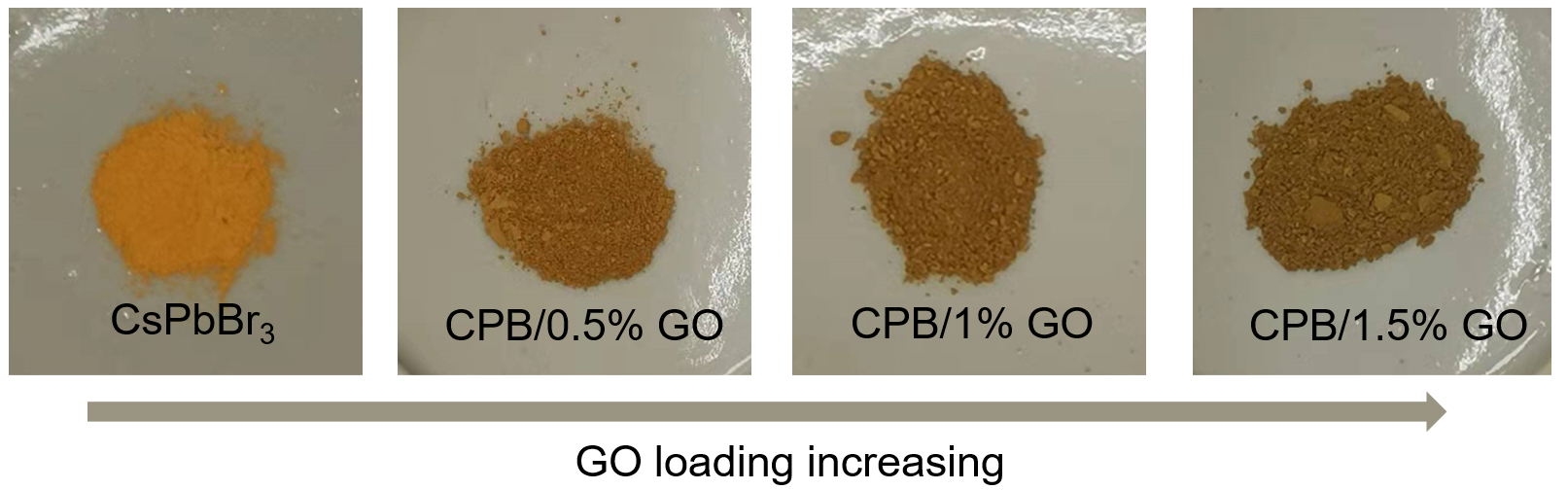


**Figure S2.** The photographs of blank CsPbBr_3_ and CPB/x% GO (x=0.5, 1, 1.5).

**Figure S3.** Tauc plots of blank CsPbBr_3_ and CPB/1% GO composite.


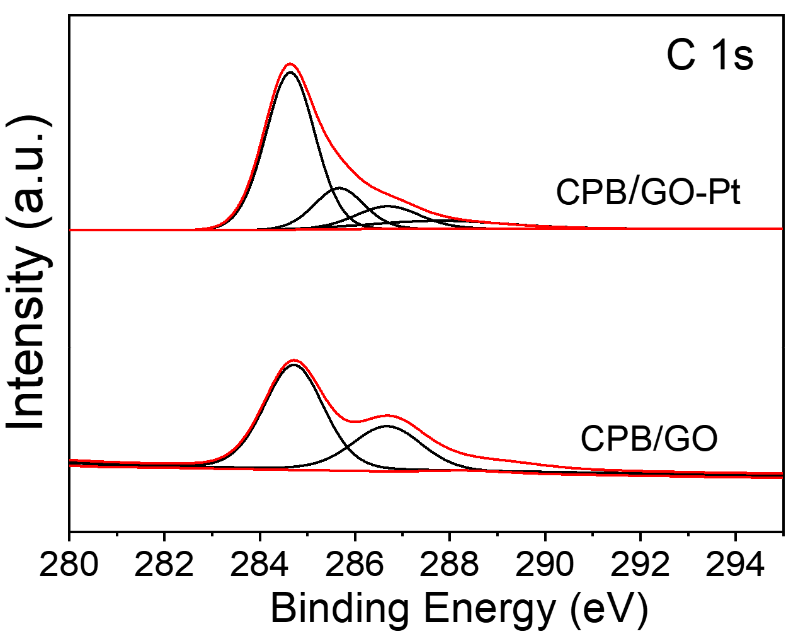


**Figure S4.** XPS spectra of C 1s in CPB/GO and CPB/GO-Pt composite.

**Table S1.** The inductively coupled plasma-mass spectrometry (ICP-MS) result of Pt content in CPB/GO-1%Pt sample.

| Sample | Theoretical value  (Pt content) | ICP-MS  (Pt content) |
| --- | --- | --- |
| CPB/GO-1%Pt | 1% | 0.95% |


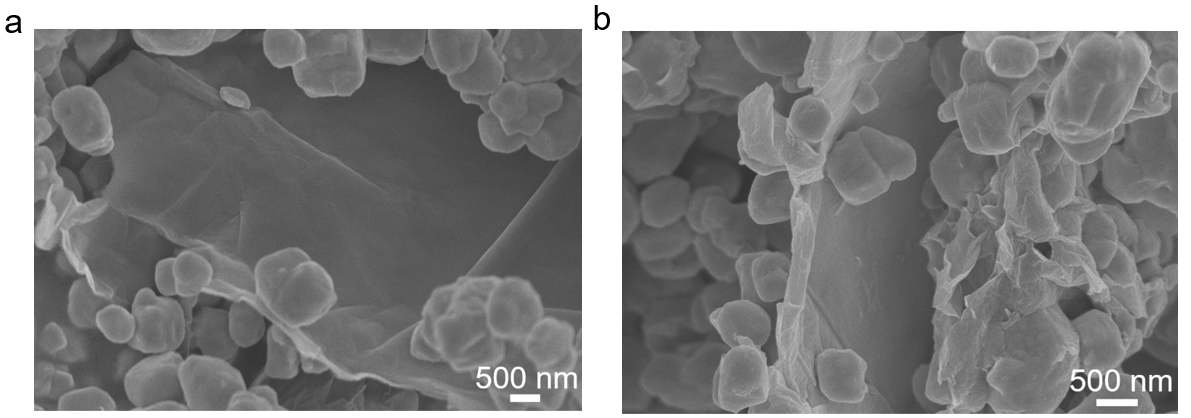


**Figure S5.** SEM images of CPB/GO-Pt composite.


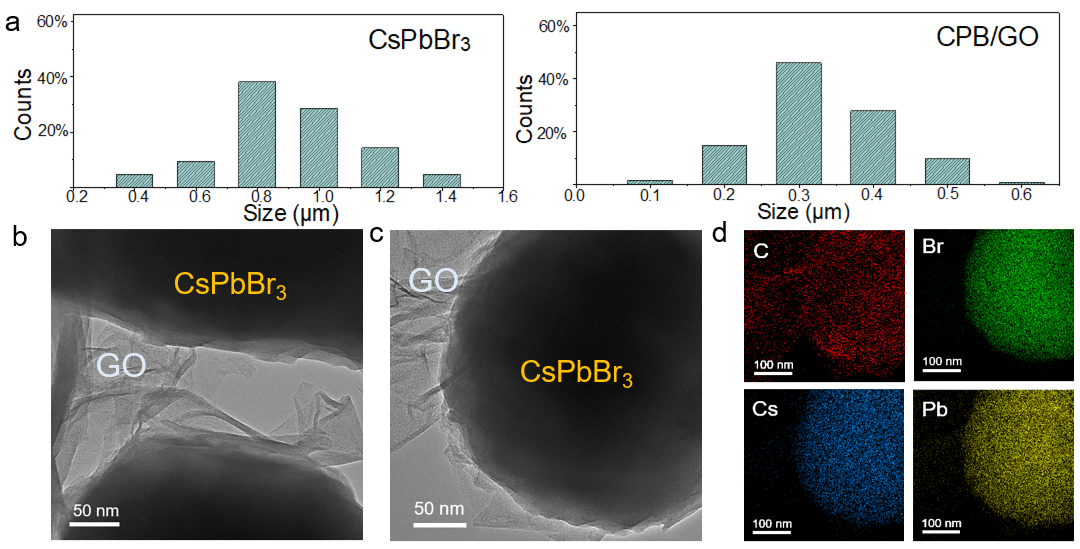


**Figure S6.** a) The size distribution of blank CsPbBr_3_ and CPB/GO composite. b, c) TEM images and d) the corresponding elemental mapping images of the CPB/GO composite.


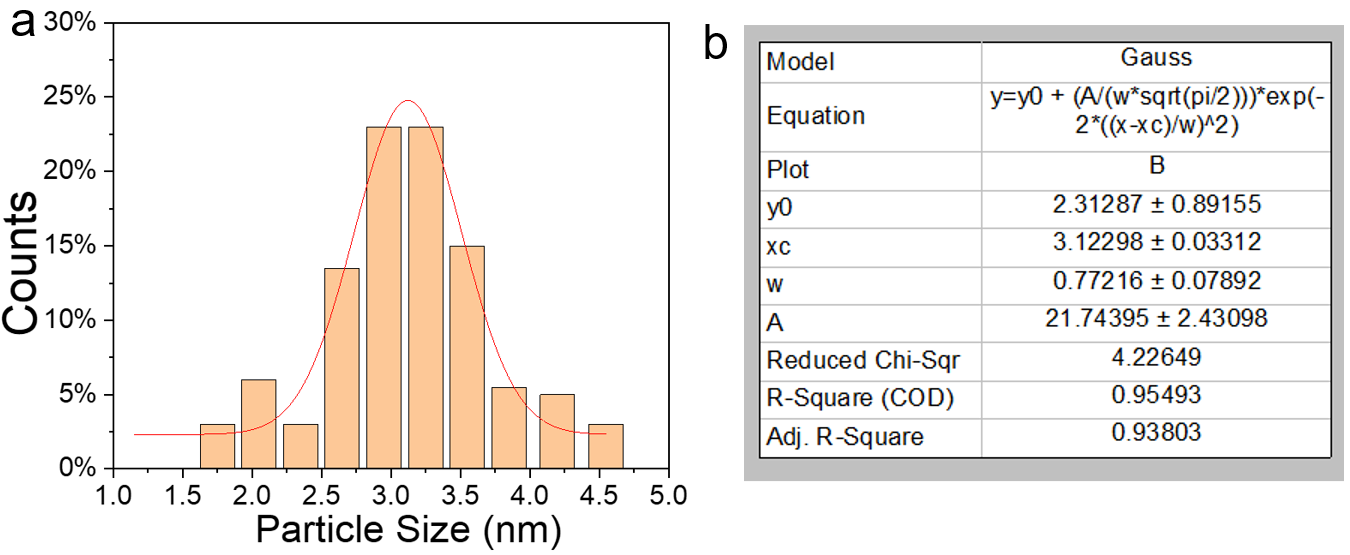


**Figure S7.** a) Size distribution of Pt nanoparticles in CPB/GO-Pt sample and b) the result of Gauss fitting.

**Note**: The particle size of Pt was measured by Nano Measurer and fitted the data to the Gaussian function. The calculated size of Pt is *ca.* 3.1 nm.

**Figure S8.** TRPL spectra of CsPbBr_3_ and CPB/GO hybrid.

**Table S2.** PL decay fitting data of CsPbBr_3_ and CPB/GO composite.

| **Catalysts** | **τ_1_ (ns)** | **A_1_(%)** | **τ_2_ (ns)** | **A_2_(%)** | **τ_ave_ (ns)** |
| --- | --- | --- | --- | --- | --- |
| CsPbBr_3_ | 1.06 | 15% | 6.14 | 85% | 5.38 |
| CPB/1% GO | 0.61 | 37% | 2.25 | 63% | 1.64 |


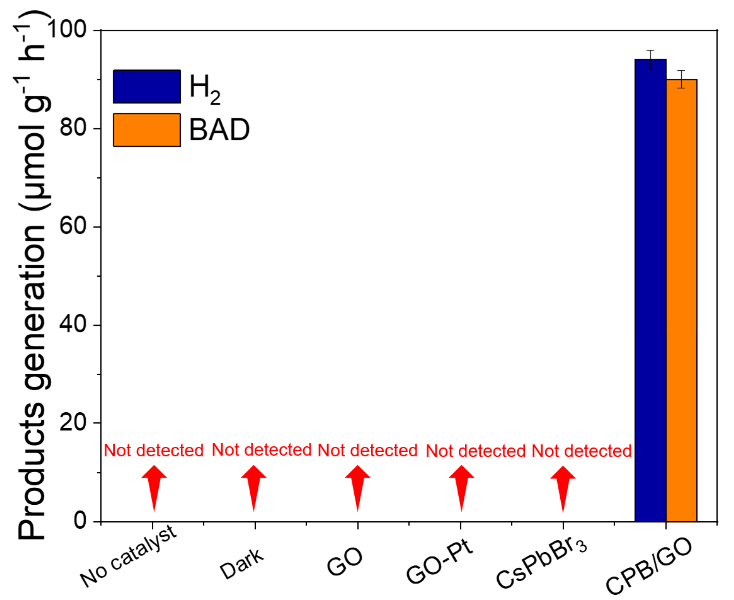


**Figure S9.** The average rates of H_2_ and BAD evolution over different samples and different test conditions. Reaction conditions: 10 mg catalyst, 0.2 mmol BA, 3 mL of CH_3_CN, Ar atmosphere, visible light (λ > 400 nm).

**Figure S10.** Long-time experiment of CPB/1.0% GO-1%Pt composite.


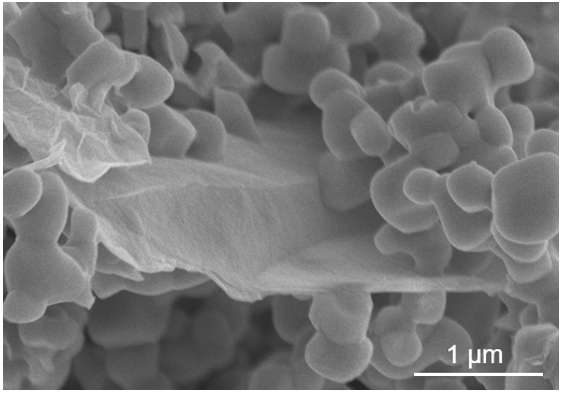


**Figure S11**. SEM image of CPB/1% GO-1%Pt after being used for 20 h.


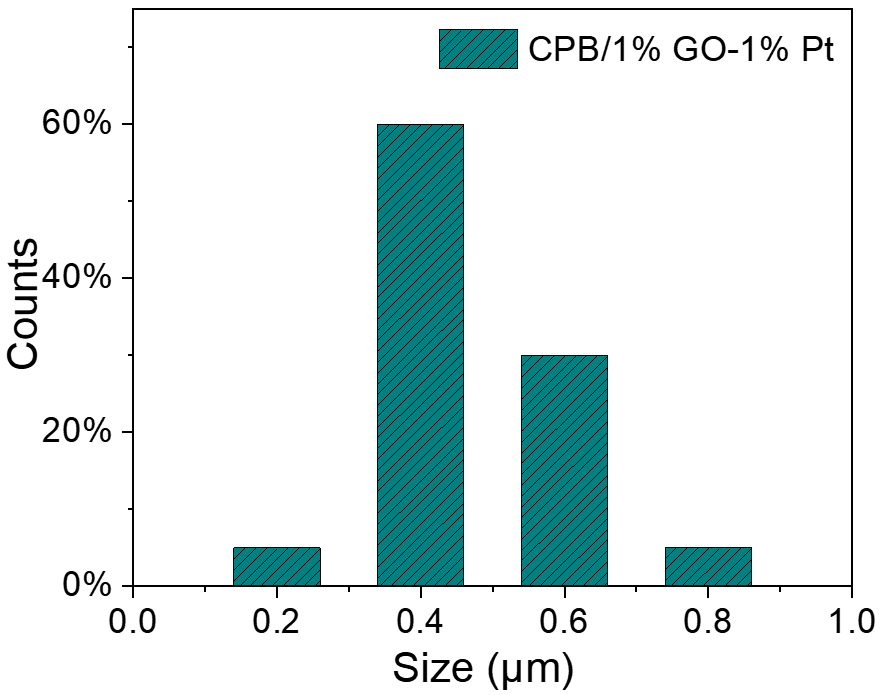


**Figure S12**. The size distribution of CPB/1% GO-1%Pt composite after being used for 20 h.


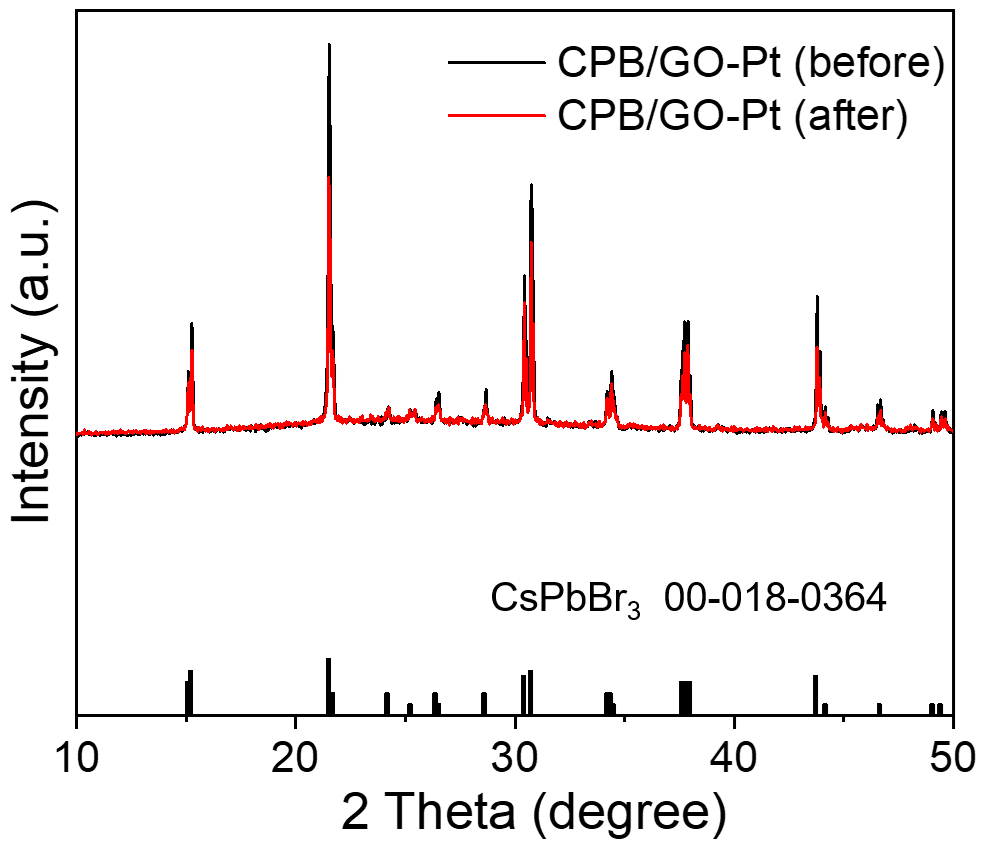


**Figure S13.** XRD pattern of CPB/GO-Pt composite before and after 20 h photo-irradiation under visible light irradiation.


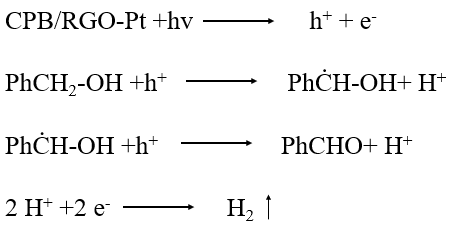


**Figure S14**. The chemical reaction equations for photocatalytic BA oxidation coupled with H_2_ generation over CPB/GO-Pt under light irradiation.
